# Temperature preconditioning is optimal at 26°C and confers additional protection to hypothermic cardioplegic ischemic arrest

**DOI:** 10.1258/ebm.2011.010357

**Published:** 2011-06

**Authors:** Igor Khaliulin, Andrew P Halestrap, M-Saadeh Suleiman

**Affiliations:** School of Biochemistry and the Bristol Heart Institute, Medical Sciences Building, University of Bristol, University Walk, Bristol BS8 1TD, UK

**Keywords:** heart, ischemia/reperfusion, hypothermia, cardioprotection, preconditioning, cardioplegia

## Abstract

We have recently shown that brief episodes of hypothermic perfusion interspersed with periods of normothermic perfusion, referred to as temperature preconditioning (TP), are cardioprotective and can be mimicked by consecutive isoproterenol/adenosine treatment. Here we investigate the optimal temperature for TP and whether TP further enhances protection provided by hypothermic ischemia with or without polarized cardioplegic arrest. Three experimental groups of Langendorff-perfused rat hearts were used. In the first group, hearts were subjected to three episodes of hypothermic perfusion at 7, 17, 26 and 32°C during the TP protocol, followed by 30 min normothermic index ischemia and 60 min reperfusion (37°C). Protein kinase A (PKA) activity and cyclic AMP (cAMP) concentrations were measured prior to index ischemia. In the second group, TP (26°C) hearts were subjected to two hours hypothermic index ischemia at 26°C and two hours normothermic reperfusion. In the third group, TP (26°C) hearts or hearts treated with isoproterenol/adenosine (pharmacological simulation of TP) were subjected to four hours hypothermic index ischemia with procaine-induced polarized cardioplegia at 26°C followed by two hours normothermic reperfusion. Hemodynamic function recovery, lactate dehydrogenase release and infarct size were used to assess cardioprotection. TP at 26°C resulted in highest cardioprotection, increased cAMP concentration and PKA activity, while TP at 7°C exacerbated ischemia/reperfusion damage, and had no effect on cAMP concentration or PKA activity. TP at 26°C also protected hearts during hypothermic ischemia with or without polarized cardioplegia. Isoproterenol/adenosine treatment conferred additional protection similar to TP. In conclusion, the study shows that TP-induced cardioprotection is temperature dependent and is optimal at 26°C; TP confers additional protection to hypothermia and polarized cardioplegia; and that the pharmacological treatment based on the mechanism of TP (consecutive isoproterenol/adenosine treatment) is a potential cardioprotective strategy that can be used during heart surgery and transplantation.

## Introduction

We have recently described a novel protocol for cardioprotection that involves subjecting the heart to brief, transient hypothermic (26°C) episodes prior to index ischemia.^1^ The choice of 26°C was based on the data of Stowe *et al.*,^2^ indicating that this is the lowest temperature that can be used without impaired contractility. Such temperature preconditioning (TP) is as least as good, if not better than ischemic preconditioning (IP) in terms of hemodynamic function, reduction in arrhythmias, oxidative stress and lactate dehydrogenase (LDH) release.^1^ We have also shown that the signaling pathway mediating TP involves activation of cyclic AMP (cAMP)-dependent protein kinase A (PKA) followed by activation of protein kinase C (PKC).^3^ A modest increase in reactive oxygen species has been found to play an important role, and an involvement of AMP-activated protein kinase (AMPK) cannot be excluded.^1^ The cardioprotective effects of TP, like IP, were associated with decreased oxidative stress at the end of index ischemia and during reperfusion which prevents opening of the mitochondria permeability transition pore (MPTP) leading to both improved contractile function and decreased necrotic damage.^1,3^

Hypothermia is routinely used in conjunction with cardioplegic solutions to protect the heart during open heart surgery. Hypothermia (25–27°C) affords considerable protection against an expected ischemic insult in patients undergoing cardiac surgery for coronary bypass grafting or valve repair.^4^ This protection is associated with better tissue perfusion, improved metabolic and mechanical function, fewer arrhythmias and reduced infarct size upon reperfusion.^5,6^ Hypothermia may mediate its protection by slowing ATP-dependent metabolism and hence mitochondrial respiration and oxidative phosphorylation.^7^ However, hypothermia also increases intracellular [Ca^2+^] ([Ca^2+^]_i_), which may be harmful to the myocardium. Increased Ca^2+^ loading during hypothermia may derive from increased net inward Ca^2+^ current flux due to markedly increased action potential duration, slowing down Ca^2+^ removal mediated by ATP-dependent pumps of the sarcolemma and sarcoplasmic reticulum.^2^ Hypothermia also diminishes Na^+^-pump activity and increases Na^+^ influx via Na^+^/H^+^ exchange^8^ leading to increased [Na^+^]_i_. This Na^+^ accumulation results in further increases in [Ca^2+^]_i_ through inhibition or reversal of the Na^+^/Ca^2+^ exchanger.^2^ Elevated [Ca^2+^]_i_ can exert deleterious effects such as hypercontracture that may jeopardize a full return of function on re-warming,^2^ eventually leading to circulatory collapse.^9^ Whether the cardioprotective efficacy of TP can also extend to hypothermic ischemic arrest is not presently known.

Along with hypothermia, cardioplegic arrest remains a popular measure to diminish ischemia-induced myocardial injury during cardiac surgery. Cardioplegic arrest during index ischemia is usually induced by a hyperkalemic extracellular solution that induces depolarization.^10^ Despite its almost universal usage, depolarized cardioplegic arrest has serious disadvantages including Na^+^ and Ca^2+^ accumulation in myocardium.^11^ This can lead to myocardial and microvascular injury, coronary vasoconstriction and spasm,^12^ arrhythmias,^13^ right and left ventricular stunning, and can potentiate the local inflammatory response.^14^ An alternative to depolarized arrest is to induce electromechanical arrest in a polarized or hyperpolarized state. This will retain ionic homeostasis during ischemia. Sodium channel blockers such as tetrodotoxin, procaine or lidocaine are able to induce a polarized arrest, considerably improving heart preservation during cardiac surgery.^11^

However, neither hypothermia nor polarized cardioplegia totally abolishes ischemia-induced myocardial damage. Thus, additional protection is needed to improve heart recovery following global ischemia in these conditions occurring during cardiac surgery. This study was aimed at determining whether TP or its pharmacological mimic (consecutive isoproterenol/adenosine treatment^3^) confer additional protection to hypothermia and polarized cardioplegic arrest. Prior to this we also sought to establish the optimal temperature for TP-induced cardioprotection.

## Methods

### Heart perfusion and analysis of hemodynamic function

All procedures conformed to the UK Animals (Scientific Procedures) Act 1986 and the Guide for the Care and Use of Laboratory Animals published by the US National Institutes of Health (NIH Publication No. 85-23, revised 1996). Ethical approval was granted by the University of Bristol, UK (Investigator number ub/09/012). Male Wistar rats (250–260 g) were killed by stunning and cervical dislocation. Hearts (∼0.75 g) were rapidly removed into ice-cold Krebs–Henseleit buffer (KH) and Langendorff heart perfusion was performed. Hemodynamic function was measured throughout all the experiments using a latex balloon in the left ventricle. Data acquisition and analysis used a PowerLab System (ADInstruments, Bella Vista, NSW, Australia). Hemodynamic left ventricular developed pressure (LVDP) was calculated as the difference between left ventricular systolic pressure (LVSP) and left ventricular end-diastolic pressure (LVEDP), and work index (rate-pressure product, RPP) as the product of LVDP and heart rate (HR). Time derivatives of pressure were measured during contraction (+d*P*/d*t*) and relaxation (−d*P*/d*t*). During ischemia, the following parameters were measured: time from the onset of ischemia to complete cessation of contractility (time to zero LVDP), time from the onset of ischemia to start of LVEDP increase due to ischemic contracture (time to ischemic contracture), and maximal LVEDP increase during ischemic contracture (maximal contracture).

### Experimental protocols

#### Experimental series 1 (Figure 1a)

After 44 min preischemia, global normothermic ischemia was induced for 30 min by halting perfusion and immersing the heart in KH at 37°C. Normothermic perfusion (37°C) was then reinstated for 60 min. Hearts were divided into five groups according to the preischemic protocol. Hearts of the Control group (*n* = 14) were not subjected to any episode of hypothermic perfusion. TP hearts experienced 20-min equilibration at 37°C and three cycles of two minutes hypothermic perfusion interspersed with six minutes normothermic perfusion. TP hearts were divided into four groups according to the temperature of brief hypothermic perfusion: TP32 (*n* = 8) – at 32°C; TP26 (*n* = 11) – at 26°C; TP17 (*n* = 10) – at 17°C and TP7 (*n* = 9) – at 7°C.

**Figure 1:**
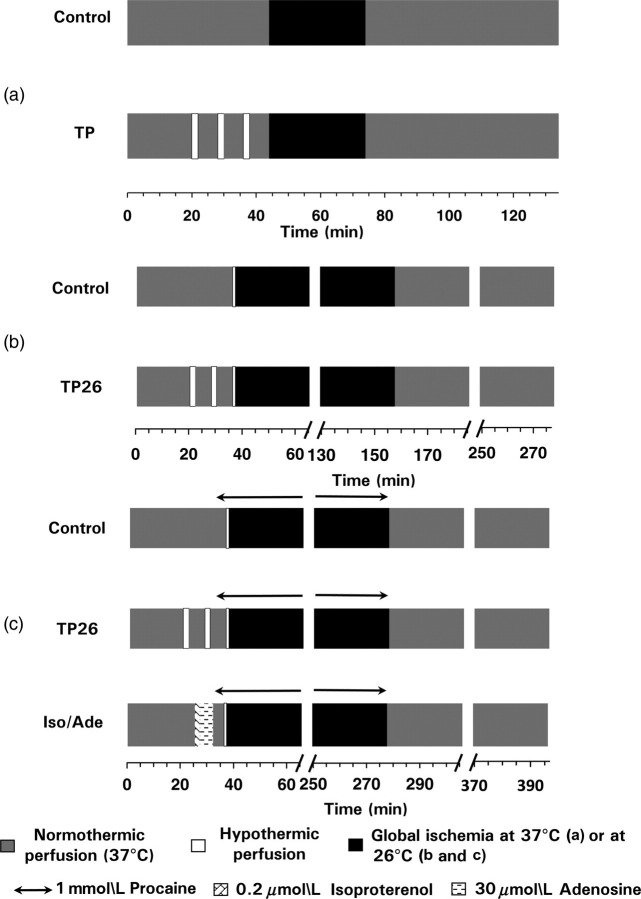
Outline of the protocols used in the experiments. (a) Experimental series 1. C – control group, TP – temperature preconditioning. TP protocol was performed at 32, 26, 17 or 7°C. Thirty minutes global ischemia and 60 min reperfusion were carried out at normothermia (37°C). (b) Experimental series 2. TP26 – TP performed at 26°C. Following the preischemic protocol, all hearts were subjected to a one-minute preischemic 26°C perfusion prior to two hours global hypothermic ischemia at 26°C. Reperfusion was carried out at normothermia and lasted for two hours. (c) Experimental series 3. TP26 – TP performed at 26°C; Iso/Ade – two minutes perfusion with 0.2 *μ*mol/L isoproterenol followed by five minutes perfusion with 30 *μ*mol/L adenosine. Polarized cardioplegia was induced by four minutes perfusion with 1 mmol/L procaine at normothermia and one minute at hypothermia (26°C) following the preischemic protocol. Global hypothermic ischemia (26°C) with cardioplegia lasted for four hours Reperfusion at normothermia without procaine lasted for two hours

#### Experimental series 2 (Figure 1b)

After 36 min preischemia, hearts were cooled by perfusing at 26°C for one minute. This brief hypothermic perfusion allows the myocardial temperature to equilibrate with the hypothermic temperature of KH buffer.^1^ Then the hearts were subjected to global hypothermic ischemia for two hours by halting perfusion and immersing the heart in perfusion buffer at 26°C. Normothermic perfusion (37°C) was reinstated after the hypothermic ischemia and lasted for two hours. Hearts were divided into two groups according to the preischemic protocol. In hearts of the Control group (*n* = 11), normothermic perfusion was carried out for 36 min. In this series of experiments, hearts of the TP26 group (*n* = 9) were subjected to two cycles of two minutes hypothermic perfusion (26°C) interspersed with six minutes normothermic perfusion instead of three cycles used in Experimental series 1. According to our preliminary experiments (data not shown), three cycles may result in LVEDP increase during reperfusion following hypothermic ischemia. Meanwhile two cycles did not affect this parameter (Figure 5a).

#### Experimental Series 3 (Figure 1c)

After 32 min preischemia, all hearts of this series were arrested by four minutes normothermic perfusion with KH buffer containing 1 mmol/L procaine (polarized cardioplegia). Hearts were then cooled by one-minute perfusion with KH buffer containing 1 mmol/L procaine at 26°C and made globally ischemic for four hours at this temperature by halting perfusion and immersing the hearts in the same buffer at 26°C. Normothermic perfusion (37°C) was subsequently reinstated for two hours with no procaine in the perfusion buffer. Hearts were divided into TP26 (*n* = 7) and Isoproterenol/adenosine (*n* = 5) groups according to the preischemic protocol and compared with 6 and 5 control hearts respectively. The protocol of TP26 was similar to that in Experimental Series 2, but after the second episode of hypothermic perfusion, these hearts were perfused with KH at 37°C for two minutes prior to normothermic perfusion with procaine. Hearts of the Isoproterenol/adenosine group were subjected to two minutes KH perfusion with 0.2 *μ*mol/L isoproterenol followed by five minutes perfusion with 30 *μ*mol/L adenosine prior to normothermic perfusion with procaine.

### Assays

Eight hearts of each of Control, TP26 and TP7 groups were freeze-clamped at the end of the experimental protocol of Series 1, ground under liquid nitrogen and stored at −80°C before the assays of cAMP and PKA.

#### cAMP

This was determined using a direct enzyme immunoassay kit (Sigma, Poole, Dorset, UK). Samples of the frozen hearts were suspended in 0.1 mol/L HCl at room temperature and centrifuged at 600***g*** for 10 min. Protein concentration in the supernatant was determined using a bicinchoninic acid-based protein assay (Pierce, Thermo Fisher Scientific, Cramlington, Northumberland, UK) and adjusted to 3 mg/mL before the spectrofluorimetric assay of cAMP according to the manufacturer's instructions.

#### PKA activity

For the assay of PKA activity, samples of the frozen and ground hearts were vortex-mixed into buffer (pH 7.4, 4°C) containing (mmol/L) 25 Tris-HCl, 0.5 EDTA, 0.5 EGTA, 10 *β*-mercaptoethanol (Sigma), and complete protease inhibitor cocktail (Roche Diagnostics, Lewis, UK) and centrifuged at 14,000***g*** for five minutes. The protein concentration in the supernatant was determined using the Biuret assay and adjusted to 4 mg/mL. PKA activity was measured using non-radioactive PepTag^®^ assays (Promega, Southampton, Hampshire, UK) that rely on a change in charge of the PepTag^®^ A1 peptide from +1 to −1 following phosphorylation. Samples (10 *μ*g in 5 *μ*L) with PKA reaction mixture (25 *μ*L) were incubated at room temperature for 15 min. After incubation, the samples were separated on a 0.8% (w/v) agarose gel at 100 V for 15–20 min. Purified PKA catalytic subunit was used as a positive control while the negative control contained only buffer. Bands were visualized under ultraviolet light and quantified using an Alpha Innotech ChemiImager 4400 with AlphaEase v5.5 software. The ratio of fluorescence intensity of phosphorylated and non-phosphorylated peptide of each sample was used as a measure of PKA activity.

#### Lactate dehydrogenase activity

Enzyme activity was determined in the effluent perfusate collected from the hearts of all groups prior to ischemia and during each five minutes over the first 15 min of reperfusion as described earlier.^15^

#### Infarct size

This was determined using triphenyltetrazolium chloride (TTC) staining as described previously.^16^ Necrotic and intact areas of each side for each of six heart slices were determined using AlphaEase v5.5 software and the total necrotic and intact area of ventricular myocardium of each heart was calculated. Since the entire heart was at risk from global ischemia, the infarct size was expressed dividing the sum of necrotic areas by the sum of total slice areas of the six slices to obtain the percentage of necrosis. Infarct size was used as an additional indicator of necrotic damage to myocardium in hearts of the Experimental series 2 and 3 where the duration of reperfusion was two hours. This duration of reperfusion is sufficient for identification of infarct size using TTC and has been used in a majority of studies on isolated rat heart.

### Statistical analysis

Data are presented as means ± SEM. Statistical significances of the differences between groups were evaluated using two-way analysis of variance (ANOVA) followed by Bonferroni *post hoc* test (hemodynamic function and LDH release), one-way ANOVA followed by Turkey's *post hoc* test (cAMP concentration and PKA activity) or Student's *t*-test (infarct size) using Graphpad Prism v5.0 software. In Series 3, data of TP26 and Isoproterenol/adenosine groups were compared with different Control groups because the experiments involving both TP26 and isoproterenol/adenosine treatment were carried out separately. Differences were considered significant where *P* < 0.05.

## Results

### Experimental series 1 – temperature-dependence of TP

#### PKA activity and [cAMP] in hearts following the different TP protocols

**Figure 2:**
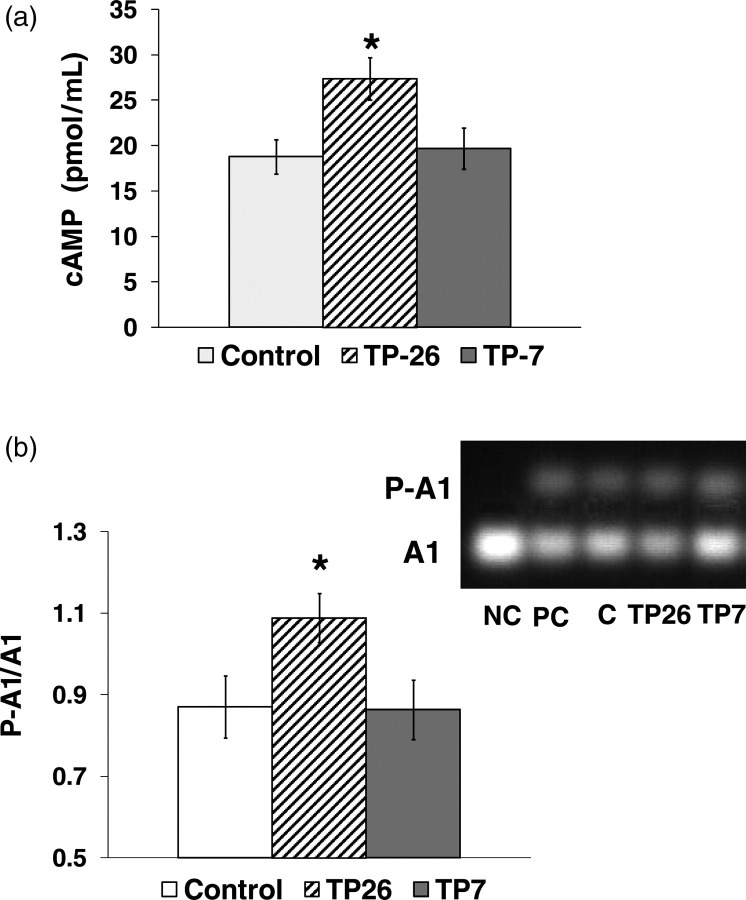
Effects of TP at 26 and 7°C on PKA activity and cAMP concentration prior to ischemia. cAMP concentration and PKA activity were determined in hearts freeze-clamped and powdered under liquid nitrogen at the end of the preischemic protocol of the experimental series 1 (Figure 1a). (a) cAMP concentration was determined using a direct enzyme immunoassay kit (Sigma). Control group of hearts; TP26 and TP7 – groups of hearts subjected to temperature preconditioning protocol at 26 and 7°C. (b) PKA activity measured using the PepTag^®^ assay (Promega) and expressed as a ratio of fluorescence intensity of phosphorylated to non-phosphorylated PepTag^®^ A1 peptide (P-A1 and A1, respectively). Inset: a representative gel containing A1 and P-A1; NC – negative control, PC – positive control (PepTag^®^ A1 peptide phosphorylated by the PKA catalytic subunit), C – control group; TP26 and TP7 – groups of hearts subjected to temperature preconditioning protocol at 26 and 7°C. *P < 0.05 versus Control. TP, temperature preconditioning; PKA, protein kinase A; cAMP, cyclic AMP

In this series of experiments, the cAMP concentration in TP26 hearts was significantly increased while in TP7 it was similar to controls (Figure 2a). Measurements of PKA activity matched cAMP concentrations: a ratio of fluorescence intensity of phosphorylated to non-phosphorylated Pep Tag^®^ A1 peptide was increased in TP26 but not in TP7 hearts (Figure 2b). Thus, TP26 hearts experienced significant PKA activation due to *β*-adrenergic stimulation, which was not observed during brief hypothermic perfusion at 7°C (TP7).

#### Hemodynamic function

**Figure 3:**
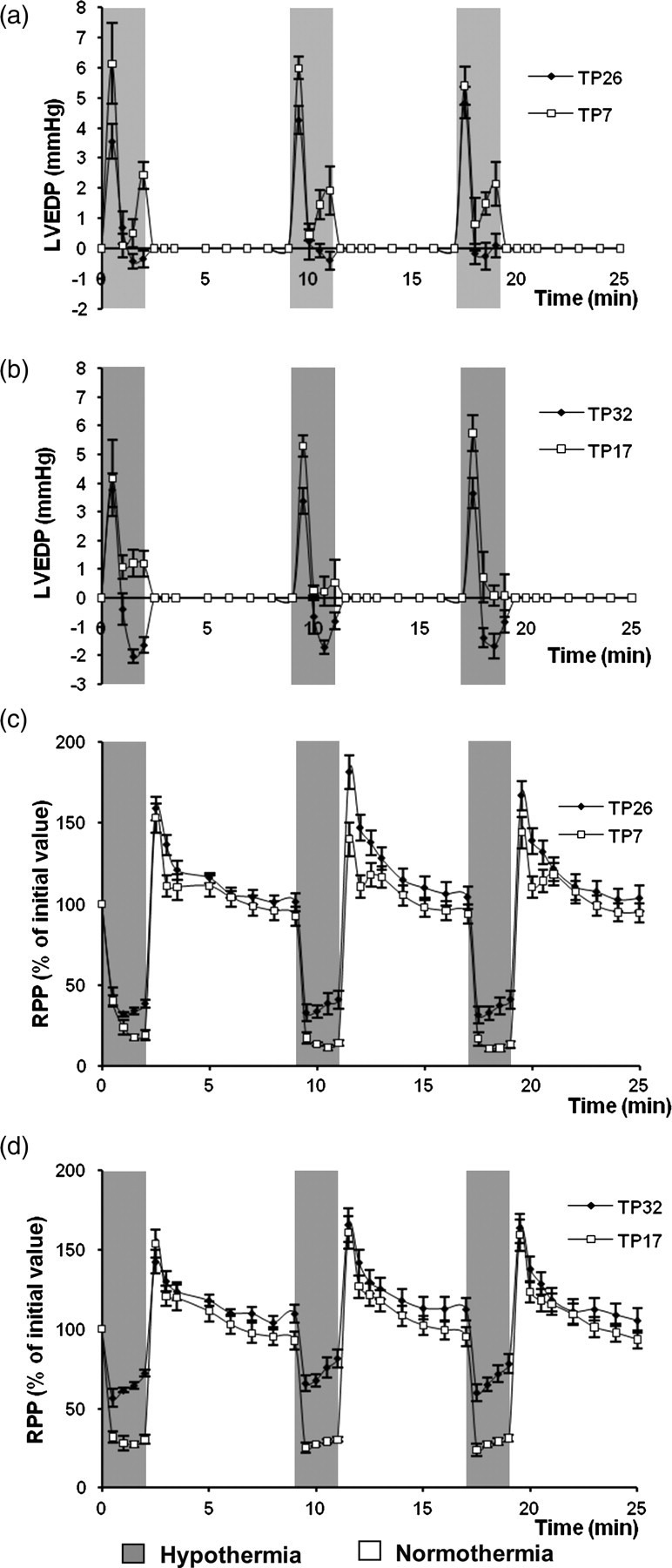
Changes of preischemic LVEDP (a,b) and RPP (c,d) during three cycles of hypothermic perfusion in TP hearts. LVEDP during hypothermia is expressed in mmHg normalized for the value of prehypothermic LVEDP; RPP is expressed as the percentage of the initial values measured at the end of the prehypothermic (equilibration) period. Hypothermic perfusions in TP26 and TP7 hearts (a and c) were performed at 26 and 7°C while hypothermic perfusions in TP32 and TP17 hearts (b and d) were performed at 32 and 17°C. LVEDP, left ventricular end-diastolic pressure; TP, temperature preconditioning; RPP, rate-pressure product

The hemodynamic parameters of control hearts did not change significantly throughout 44 min preischemic perfusion. In all groups of TP hearts, hypothermic perfusion during TP resulted in a considerable decrease of RPP, mostly due to reduced HR. Diastolic pressure was elevated during two minutes hypothermic perfusion. Subsequent restoration of normothermia led to rapid augmentation of contractile function, mainly as a result of increased LVDP. However, some significant differences between the TP groups were apparent during preischemia (Figure 3). At 32 and 26°C, the initial short-term increase of diastolic pressure was followed by a fall to below the initial value. At lower temperatures (17 and 7°C), the initial increase in diastolic pressure was much higher, but here a secondary rise was observed during episodes of hypothermia. This was greatest at 7°C (Figures 3a and b). Figures 3c and d compare the changes of RPP during periods of perfusion at 32, 26, 17 and 7°C. These data reveal that it was during the second episode of normothermic perfusion that the maximal increase of LVDP and RPP was greater in the TP26 hearts than TP7 hearts.

In all groups of hearts, global normothermic ischemia progressively reduced LVDP until complete cessation of hemodynamic function was achieved, while diastolic pressure increased later during ischemia, reflecting development of ischemic contracture. However, TP26 hearts showed the shortest time to zero LVDP, the longest delay before ischemic contracture began and the lowest magnitude of ischemic contracture similar to the changes of these parameters reported previously.^1^ Time to start of ischemic contracture was also significantly longer in TP17 than control hearts (13.1 ± 0.5 and 11.4 ± 0.5 min respectively, *P* < 0.05), while in TP32 and TP7 hearts these parameters were not significantly different from control hearts.

**Figure 4:**
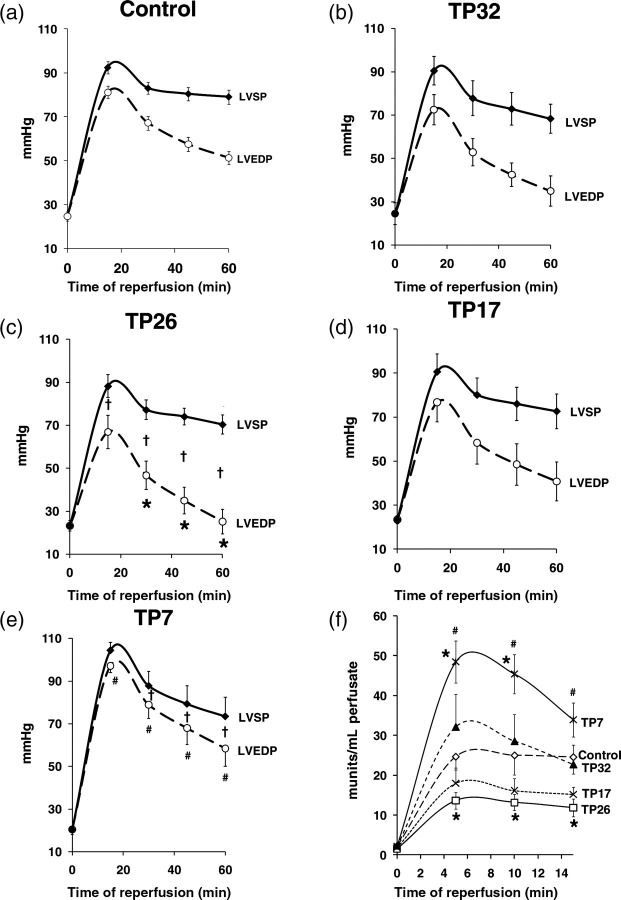
Hemodynamic function recovery and LDH release during reperfusion after 30 min global normothermic ischemia. Panels a–e present changes of LVEDP, LVSP and LVDP (difference between LVSP and LVEDP) during 60 min normothermic reperfusion after 30 min global normothermic ischemia in different groups of hearts of the experimental series 1 (Figure 1a). (f) LDH activity measured in the effluent perfusate during the first 15 min reperfusion in the groups of hearts of the experimental series 1. *P < 0.05 versus Control; ^#^ *P* < 0.05 versus TP26. There was no difference in heart rate recovery between the groups and the symbol † indicates statistically significant differences (*P* < 0.05) in both LVDP and RPP versus Control. LVEDP, left ventricular end-diastolic pressure; LVSP, left ventricular systolic pressure; LVDP, left ventricular developed pressure; LDH, lactate dehydrogenase; RPP, rate-pressure product

During reperfusion, recovery of hemodynamic function was greatest in TP26 hearts with recovery of LVDP being twice as high as for control hearts. TP32 and TP17 hearts did not show any improvement in hemodynamic recovery while TP7 hearts exhibited a further deterioration in function compared with controls (Figures 4a–e). HR during reperfusion did not differ from its preischemic value in any group of hearts. Thus recovery of RPP (a product of LVDP and HR) during reperfusion largely depended on the recovery of LVDP.

#### Necrotic damage during reperfusion (LDH release)

This followed a similar pattern to the recovery of hemodynamic function (Figure 4f) with the TP26 group showing a considerably reduced LDH release whereas the TP7 group showed increased LDH release compared with controls.

### Experimental series 2 – cardioprotection by TP during prolonged hypothermic ischemia

Hypothermic ischemia prolonged the time to zero LVDP four-fold, increased time to onset of ischemic contracture and reduced the amplitude of maximal contracture two-fold compared with normothermic ischemia. However, TP26 showed a shorter time from the onset of hypothermic ischemia to zero LVDP (8.9 ± 0.4 in TP26 group versus 13.7 ± 0.9 min in the Control group, *P* < 0.05) and 36% less ischemic contracture compared with control (15.6 ± 1.8 and 24.4 ± 3.1 mmHg, respectively, *P* < 0.05), consistent with these parameters measured during normothermic ischemia.

**Figure 5:**
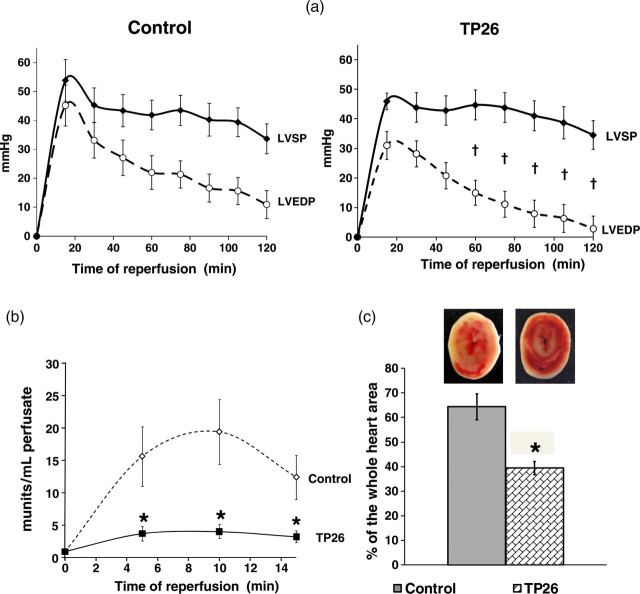
Hemodynamic function recovery, LDH release and infarct size after two hours global hypothermic ischemia and two hours reperfusion. (a) Changes of LVEDP, LVSP and LVDP (difference between LVSP and LVEDP) during two hours normothermic reperfusion after two hours global hypothermic ischemia (26°C) in the groups of hearts of the experimental series 2 (Figure 1b). (b) LDH activity measured in the effluent perfusate during the first 15 min reperfusion in the groups of hearts of the experimental series 2. (c) Infarct size measured at the end of two hours normothermic reperfusion in the groups of hearts of the experimental series 2. Infarct size is expressed as mean ± SEM of the percentage of necrotic area relatively to the whole heart area. TP26 – group of hearts subjected to temperature preconditioning protocol at 26°C. Inset: corresponding representative images of the heart slices. *P < 0.05 versus Control. There was no difference in heart rate recovery between the groups and the symbol † indicates statistically significant differences (*P* < 0.05) in both LVDP and RPP versus Control. LVEDP, left ventricular end-diastolic pressure; LVSP, left ventricular systolic pressure; LVDP, left ventricular developed pressure; LDH, lactate dehydrogenase; RPP, rate-pressure product

During reperfusion, TP26 hearts showed a significantly improved recovery of hemodynamic function (Figure 5a) and LDH release appeared to be five-fold lower than control at 10-min of reperfusion (Figure 5b). LDH release paralleled the infarct size measurements (Figure 5c): necrotic areas in control and TP26 hearts were 64.3 ± 2.3% and 39.5 ± 2.2 5% of the whole heart area, respectively (*P* < 0.001).

### Experimental series 3 – cardioprotection by TP or isoproterenol/adenosine treatment during prolonged hypothermic ischemia with polarized cardioplegia

**Figure 6:**
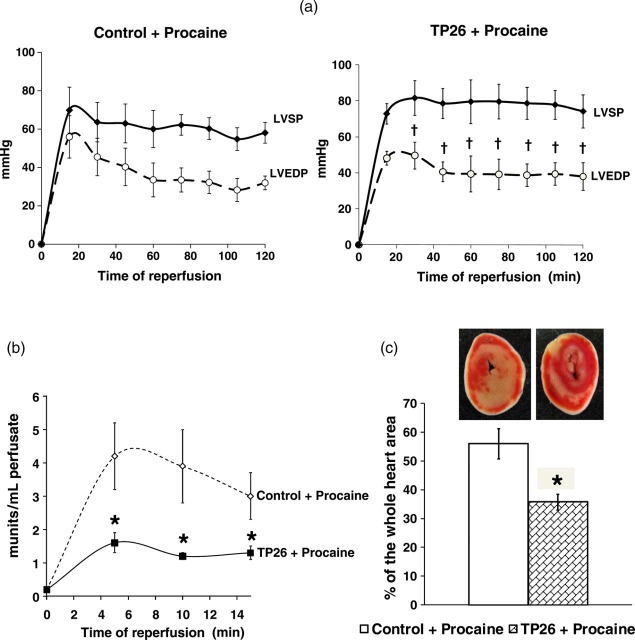
Hemodynamic function recovery, LDH release and infarct size after four hours global hypothermic ischemia and polarized cardioplegia and two hours reperfusion in TP26 hearts. (a) Changes of LVEDP, LVSP and LVDP (difference between LVSP and LVEDP) during two hours normothermic reperfusion after four hours global hypothermic ischemia (26°C) and polarized cardioplegia, induced by 1 mmol/L procaine, in the Control and TP26 groups of hearts of the experimental series 3 (Figure 1c). (b) LDH activity measured in the effluent perfusate during the first 15 min reperfusion in the Control and TP26 groups of hearts of the experimental series 3. (c) Infarct size measured at the end of two hours normothermic reperfusion in the Control and TP26 groups of hearts of the experimental series 3. Infarct size is expressed as mean ± SEM of the percentage of necrotic area relatively to the whole heart area. Inset: corresponding representative images of the heart slices. *P < 0.05 versus Control. There was no difference in heart rate recovery between the groups and the symbol † indicates statistically significant differences (*P* < 0.05) in both LVDP and RPP versus Control. LVEDP, left ventricular end-diastolic pressure; LVSP, left ventricular systolic pressure; LVDP, left ventricular developed pressure; LDH, lactate dehydrogenase; RPP, rate-pressure product

**Figure 7:**
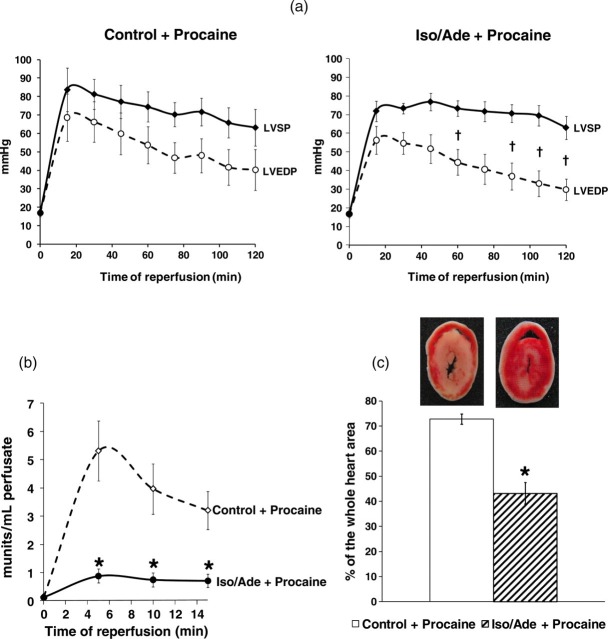
Hemodynamic function recovery, LDH release and infarct size after four hours global hypothermic ischemia and polarized cardioplegia and two hours reperfusion in hearts treated consecutively with isoproterenol and adenosine (Iso/Ade group). (a) Changes of LVEDP, LVSP and LVDP (difference between LVSP and LVEDP) during two hours normothermic reperfusion after four hours global hypothermic ischemia (26°C) and polarized cardioplegia, induced by 1 mmol/L procaine, in Control and Iso/Ade groups of hearts of the experimental series 3 (Figure 1c). (b) LDH activity measured in the effluent perfusate during the first 15 min reperfusion in the Control and Iso/Ade groups of hearts of the experimental series 3. (c) Infarct size measured at the end of two hours normothermic reperfusion in the Control and Iso/Ade groups of hearts of the experimental series 3. Infarct size is expressed as mean ± SEM of the percentage of necrotic area relatively to the whole heart area. Inset: corresponding representative images of the heart slices. *P* < 0.05 versus Control. There was no difference in heart rate recovery between the groups and the symbol † indicates statistically significant differences (*P* < 0.05) in both LVDP and RPP versus Control. LVEDP, left ventricular end-diastolic pressure; LVSP, left ventricular systolic pressure; LVDP, left ventricular developed pressure; LDH, lactate dehydrogenase; RPP, rate-pressure product

Prior to ischemia, perfusion with 1 mmol/L procaine caused heart contraction to stop after 2.3 ± 0.2, 2.2 ± 0.2 and 2.4 ± 0.3 min in control, TP26 and isoproterenol/adenosine hearts, respectively (*P* > 0.05). Hearts not treated with procaine were unable to recover after four hours hypothermic ischemia (26°C) (data not shown) while procaine prevented any contracture during four hours hypothermic ischemia and enabled hearts to recover partially during reperfusion. However, both TP26 and isoproterenol/adenosine treatments were still able to improve hemodynamic function recovery and reduce LDH release during reperfusion in hearts arrested with hypothermic cardioplegia (Figures 6a, b and 7a, b). The data of Figures 6c and 7c show that infarct size was also significantly less in isoproterenol/adenosine hearts compared with controls (43.2 ± 4.3% versus 72.8 ± 2.0% of the whole heart area, *P* < 0.01) as it was for TP26 hearts (35.7 ± 2.7% versus 56.0 ± 5.3% of the whole heart area, *P* < 0.01).

## Discussion

### TP is optimal at 26°C

The data presented here demonstrate that the protective effects of TP are critically dependent on the temperature of the short-term episodes of hypothermic perfusion, with 26°C giving the best protection. During global normothermic ischemia, hearts of the TP26 group showed the shortest time to zero LVDP, the longest time to start of ischemic contracture and the lowest increase of diastolic pressure during ischemic contracture. We have previously shown that these effects reflect better preservation of high-energy phosphates in the TP26 hearts.^1^ These hearts also had the highest hemodynamic function recovery and the lowest LDH release during reperfusion (Figure 4) indicating the lowest necrotic damage to the myocardium. TP at either lower (17°C) or higher (32°C) temperatures was not as protective, while the use of 7°C actually exacerbated reperfusion injury and was associated with lower hemodynamic recovery and higher LDH release than in the control hearts.

One reason for the deleterious effect of TP at 7°C may be an excessive accumulation of calcium in the myocardium as a result of an imbalance between active and passive ion membrane transport.^2,17^ During hypothermia, ATP-dependent ion channels become inhibited while Na^+^, Ca^2+^ and K^+^ diffusion are less affected. An imbalance between the activity of the ATP-dependent Ca^2+^ pump of sarcoplasmic reticulum and the ryanodine receptors may also contribute to intracellular Ca^2+^ accumulation during hypothermia as may the activity of the Na^+^/Ca^2+^ exchanger. The severity of this imbalance depends on temperature. Thus, it has been shown that moderate hypothermia reduces membrane-bound Ca^2+^-pump activity while severe hypothermic storage abolishes it.^18,19^ Stowe *et al.* showed that at 27°C, paced hearts adapt functionally to the greater accumulation of Ca^2+^ during diastole by reducing the rate of release of Ca^2+^. However, at 17°C, despite the very prolonged release and re-uptake of Ca^2+^ over the cardiac cycle, diastolic Ca^2+^ loading becomes so marked that relaxation is impaired, and contractility decreases.^2^ The dependence of the LVEDP on the temperature of the hypothermic episodes during TP (Figure 3) is consistent with this. Thus, the lowest LVEDP was found in TP32 hearts while the highest value of this parameter was in TP7 hearts (Figures 3a and b). The increased diastolic pressure can be explained, at least partially, by a higher accumulation of intracellular Ca^2+^ at greater hypothermia. This hypothesis can be supported by the observation that rapid cooling of the myocardium to 1°C causes contracture as a result of calcium release from sarcoplasmic reticulum.^20^ It seems likely that at 7°C, Ca^2+^ accumulation is substantially greater than at 26 and 17°C and contributes to the greater ischemia/reperfusion damage.^21^ However, moderate intracellular calcium accumulation before prolonged ischemia has been reported to be protective^22,23^ and may play a role in the cardioprotective effects of TP, which our data suggest is optimal at 26°C.

The considerable rise of LVDP and RPP during re-warming (Figures 3c and d) may also be partially explained by Ca^2+^ accumulation in cardiomyocytes during hypothermia. However, it may also involve PKA activation by the release of endogenous catecholamines during hypothermia, which in turn may modulate Ca^2+^ homeostasis. Indeed, TP at 26°C (TP26) significantly increased cAMP concentration and PKA activity leading to cardioprotection during ischemia and reperfusion (see^3^ and Figures 2a and b). Interestingly, we found no difference in these parameters in TP7 hearts compared with controls (Figures 2a and b). This finding is consistent with a higher RPP elevation in TP26 hearts compared with TP7 hearts after the second short-term episode of hypothermic perfusion (Figure 3c). It would appear that severe and/or prolonged hypothermia, if not supported by other protective measures, switches off the mechanism(s) responsible for the myocardial response to *β*-adrenergic stimulation. This would affect the ability of the heart to regain its contractility during re-warming and also disrupt the signal transduction pathways of TP, thus explaining why TP7 hearts experienced greater ischemia/reperfusion damage than control hearts. By contrast, short-term episodes of hypothermic perfusion at ∼26°C stimulate this *β*-adrenergic response, enabling hearts to survive better during prolonged ischemia.^3^

### TP protects hearts against prolonged hypothermic ischemia

A number of studies have demonstrated the ability of IP to protect the heart against prolonged hypothermic ischemia, which may occur during cardiac surgery and heart transplantation.^24,25^ In these experiments, mild to moderate hypothermia (32–17°C) was employed. However, others have shown no protection when IP hearts were stored at lower temperature (6–8°C).^26^ Thus, the ability of IP to contribute to cardioprotection during hypothermic ischemia is temperature dependent. There are some similarities in the signaling mechanisms of IP and TP,^1,3^ suggesting that TP might confer additional protection to hypothermic preservation of hearts as does IP. The nature of the TP protocol might make it more effective than IP in providing such protection and our data confirm this to be the case. TP26 hearts showed significantly improved hemodynamic function recovery, and dramatically reduced LDH release and infarct size after two hours ischemia at 26°C (Figure 5). Hypothermia reduced both the speed of decline of LVSP during ischemia and the extent of ischemic contracture. Yet even under these conditions, TP26 was still able to shorten the time to zero LVDP and reduce ischemic contracture. As the key cause of ischemic contracture is lack of MgATP,^27^ it is possible that the TP26 protocol enhances ATP preservation during ischemia at 26°C over and above the energy sparing effect of hypothermia,^28^ just as we observed during normothermic ischemia.^1^ Depletion of ATP during ischemia together with oxidative stress during reperfusion promotes opening of the MPTP that ultimately causes irreversible damage to myocardium.^29^ We have recently shown that TP26 strongly inhibits MPTP opening during reperfusion^1,3^ and this might be explained, at least partially, by its ability to preserve myocardial ATP levels.

### TP enhances cardioprotection by hypothermic polarized cardioplegia

Cardioplegic arrest using a hyperkalemic extracellular solution remains the current gold standard for cardioprotection during cardiac surgery.^10,11^ Hyperkalemia induces arrest by establishing a new resting membrane potential which is at a more positive value (i.e. is depolarized from normal) and is, therefore, termed ‘depolarized’ arrest. However, it has been shown that the Na^+^ ‘window’ current during depolarized cardioplegia would lead to increased intracellular [Ca^2+^] through the calcium ‘window’ current.^30^ High [Ca^2+^]_i_ may also be caused by calcium influx through the slow calcium channel and reversed activity of the Na^+^/Ca^2+^ exchanger.^11^ This would lead to contracture, calcium overload and subsequent myocardial injury, particularly during reperfusion. A potential way to address this problem would be to induce a polarized arrest at membrane potentials closer to the normal ‘resting’ membrane potential. Agents with local anesthetic effects (such as procaine and lidocaine) are available clinically and have been widely used either alone or in combination with other agents to induce cardiac arrest.^31^ Their beneficial effects include reduction of ionic imbalance leading to reduced energy utilization, and hence improved protection from a cellular perspective.^10^ However, the protective effect of polarized cardioplegia is also time-limited. Thus in our experiments, hemodynamic recovery of control hearts, arrested prior to four hours global hypothermic ischemia (26°C) with 1 mmol/L procaine, was very poor. These data confirm the observation that cardiac surgery continues to be limited by an inability to achieve complete myocardial protection from ischemia–reperfusion injury despite the use of hypothermia and cardioplegic arrest.^32^ Furthermore, a number of studies indicate very restricted^24^ or no ability of IP to improve heart recovery after cardioplegic arrest.^33^ However, TP26 was able to improve hemodynamic function recovery and significantly reduce necrotic damage under these conditions (Figure 6). Therefore, a pharmacological treatment based on the mechanisms of TP could be beneficial when using polarized cardioplegia for hypothermic heart preservation during cardiac surgery and transplantation. We have recently shown that the key mechanism of TP is the consecutive PKA and PKC activation. We induced activation of these kinases pharmacologically, using a non-selective *β*-adrenergic agonist isoproterenol and a PKC activator adenosine. This consecutive isoproterenol/adenosine treatment protected the isolated rat heart against normothermic global ischemia very effectively.^3^ Here we demonstrate that such consecutive treatment can also protect the heart against prolonged ischemia during hypothermia and polarized cardioplegic arrest just as effectively as TP. Because pharmacological treatment is easier to perform than short-term episodes of hypothermia during cardiac surgery, this treatment may provide an intervention that will help to protect hearts in these conditions yet has much greater clinical potential than TP.

In conclusion, the novelty of our study is as follows:

1. Choice of the hypothermic temperature is critical to the cardioprotective effects of TP; 26°C is optimal for the TP protocol while temperatures below 17°C afford no cardioprotection and can be detrimental. This fact may be taken into consideration when choosing the level of hypothermia during surgery;
2. Pharmacological signaling associated with *β*-adrenergic stimulation during TP and cardioprotection is temperature dependent;
3. TP at 26°C confers additional protection to hypothermic ischemia and to hypothermic polarized cardioplegia, in contrast to IP, which affords little additional protection to cardioplegic arrest;
4. Pharmacological mimics of TP (isoproterenol/adenosine treatment) prior to hypothermic ischemic cardioplegic arrest may provide clinical benefit during heart surgery and transplantation.

**Author contributions:** All authors participated in the design, interpretation of the data and review of the manuscript. IK conducted the experiments and wrote the manuscript with input from M-SS and APH.
